# Orthostatic Hypertensive Emergency in Obesity-Related Heart Failure With Preserved Ejection Fraction (HFpEF): A Single-Center Retrospective Observational Study

**DOI:** 10.7759/cureus.111494

**Published:** 2026-06-25

**Authors:** Laya Anand, Trevor Lovell, Sudha Arulalan, Frank Annie, Adam Belcher, Chance L Pettry, Elie Gharib, Smrita Dorairajan, Anand Chockalingam

**Affiliations:** 1 Cardiology, Charleston Area Medical Center, Charleston, USA; 2 Statistics, Charleston Area Medical Center, Charleston, USA; 3 Biostatistics and Epidemiology, Charleston Area Medical Center, Charleston, USA; 4 Nephrology, Harry S. Truman Memorial Veterans' Hospital, Columbia, USA

**Keywords:** cardiometabolic, cardiometabolic complications, cardiometabolic diseases, heart failure with preserved ejection fraction (hfpef), hypertension emergency, lifestyle intervention, obesity-related illnesses, orthostatic hypertension (oht)

## Abstract

Background: Orthostatic hypertension is an under-recognized abnormal blood pressure phenotype defined by a paradoxical increase in systolic blood pressure after standing. Consensus definitions describe an exaggerated orthostatic pressor response as a systolic blood pressure increase of at least 20 mmHg after standing, with orthostatic hypertension present when standing systolic blood pressure reaches at least 140 mmHg. Although orthostatic hypertension has been associated with adverse cardiovascular and cerebrovascular outcomes, acute symptomatic presentations resembling a hypertensive emergency remain poorly characterized.

Objective: This study describes the clinical characteristics, hemodynamic profile, management, and follow-up outcomes of hospitalized patients with symptomatic orthostatic hypertensive emergency in the setting of severe obesity and obesity-related heart failure with preserved ejection fraction (HFpEF).

Methods: We conducted a single-center retrospective observational study of hospitalized adult patients evaluated by cardiology who had orthostatic blood pressure measurements obtained during routine clinical care. Patients were included if they had an orthostatic systolic blood pressure increase of at least 20 mmHg from supine to standing, a standing systolic blood pressure of at least 140 mmHg, and associated neurological or cardiac symptoms. Patients were excluded if they were critically ill, unable to stand, or receiving vasopressor therapy or lacked complete orthostatic blood pressure documentation. Demographics, comorbidities, symptoms, blood pressure values, echocardiographic findings, cardiac catheterization results when available, treatment strategies, lifestyle adherence, weight change, and follow-up outcomes were abstracted from the medical record.

Results: Seven patients met the inclusion criteria. The median age was 56 years, and body mass index ranged from 44.1 to 78.2 kg/m². Initial orthostatic systolic blood pressure increases ranged from 21 to 44 mmHg, with a peak standing systolic blood pressure ranging from 148 to 194 mmHg at index presentation. One patient later developed recurrent severe orthostatic hypertension, with systolic blood pressure increasing from 109 mmHg supine to 188 mmHg standing. Presenting symptoms included dyspnea, chest pain, pulmonary edema, syncope or near-syncope, confusion, and stroke-like symptoms. All patients had severe obesity and clinical features consistent with obesity-related cardiometabolic disease and HFpEF. Patients who adhered to structured cardiometabolic lifestyle and medical therapy demonstrated weight loss and improvement or resolution of orthostatic symptoms and blood pressure abnormalities. Patients who were non-adherent experienced persistent symptoms, recurrent hospitalization, or loss to follow-up.

Conclusion: Orthostatic hypertensive emergency may represent an under-recognized acute phenotype of postural blood pressure dysregulation in patients with severe obesity, cardiometabolic disease, and HFpEF. Routine orthostatic blood pressure assessment in hospitalized patients with obesity-related heart failure symptoms may improve the recognition of clinically significant blood pressure variability. The very small sample size of our experience serves as a hypothesis-generating report. Larger prospective studies are needed to define prevalence, prognosis, mechanisms, and optimal treatment strategies.

## Introduction

Orthostatic hypertension is increasingly recognized as a clinically meaningful blood pressure (BP) phenotype characterized by an abnormal rise in systolic BP after standing. The American Autonomic Society and Japanese Society of Hypertension consensus statement defines an exaggerated orthostatic pressor response as an increase in systolic BP of at least 20 mmHg when moving from the supine to standing position while reserving the term orthostatic hypertension for cases in which upright systolic BP is at least 140 mmHg [1]. This definition provides a standardized framework for identifying patients with clinically relevant postural BP elevation.

Orthostatic hypertension has been associated with target organ damage, progression to sustained hypertension, cardiovascular events, and cerebrovascular disease [2,3]. A systematic review and meta-analysis found that systolic orthostatic hypertension was associated with higher all-cause mortality, higher cardiovascular mortality, and increased odds of stroke or cerebrovascular disease [3]. Despite these associations, orthostatic hypertension remains less familiar in clinical practice than orthostatic hypotension and is often not systematically assessed in hospitalized patients.

The clinical relevance of orthostatic hypertension may be particularly important in patients with obesity-related heart failure with preserved ejection fraction (HFpEF). HFpEF is increasingly linked to obesity, sedentariness, hypertension, metabolic disease, systemic inflammation, autonomic dysregulation, and impaired vascular reserve [4,5]. Obesity-related HFpEF is now recognized as a distinct phenotype with unique diagnostic and therapeutic challenges [5]. In this population, abrupt postural BP elevation may worsen myocardial workload, pulmonary congestion, cerebral symptoms, and functional limitation.

We aim to describe in this single-center retrospective observational study of hospitalized patients the clinical characteristics, hemodynamic profile, management, and follow-up outcomes of marked orthostatic systolic BP elevation. Our intention is to use this small case series to generate a hypothesis for characterizing prevalence in patients with severe obesity and HFpEF presenting with acute cardiac or neurological symptoms. We suggest using the term orthostatic hypertensive emergency to describe this symptomatic phenotype, defined by orthostatic hypertension accompanied by acute cardiac or neurological symptoms concerning for end-organ stress.

## Materials and methods

Study design and setting

We performed a single-center retrospective observational study of hospitalized adult patients evaluated by the cardiology service at Charleston Area Medical Center (CAMC) in Charleston, West Virginia. Patients were identified through a retrospective review of clinical encounters in which orthostatic vital signs were obtained during routine inpatient cardiovascular assessment.

Study population

Orthostatic BP measurements were obtained during routine inpatient care by nursing or clinical staff using calibrated noninvasive automated oscillometric BP devices available on the inpatient units. Measurements were abstracted from the electronic medical record. The standard clinical sequence consisted of a supine BP measurement after the patient had been resting in bed, followed by a standing BP measurement obtained after approximately three minutes of standing, when tolerated. Patients were positioned supine for the baseline measurement and standing upright for the postural measurement. Only patients with documented paired supine and standing BP values were included. Repeat orthostatic measurements were not performed according to a uniform research protocol and were available only when obtained for clinical reasons. Medication administration status at the exact time of orthostatic assessment, including timing relative to antihypertensive or diuretic dosing, was not standardized and was abstracted when documented. Orthostatic BP measurement was not standardized. Timing of medications and BP measurement, cuff size, protocol for patient rest duration, diet, and repeat measurements are needed for reproducibility. Lack of this is a major limitation of this study.

Inclusion criteria

Patients were included if they met all of the following criteria: (1) orthostatic systolic BP increase of at least 20 mmHg from supine to standing, (2) standing systolic BP of at least 140 mmHg, (3) associated cardiac or neurological symptoms, including dyspnea, chest pain, pulmonary edema, syncope or near-syncope, confusion, altered mental status, dizziness, or stroke-like symptoms, and (4) clinical features consistent with severe obesity, cardiometabolic disease, or obesity-related HFpEF.

Exclusion criteria

Patients were excluded if they had a critical illness preventing reliable orthostatic measurement, an inability to stand, active vasopressor use, or incomplete orthostatic BP documentation.

Study definition

For this study, orthostatic hypertensive emergency was defined as an orthostatic systolic BP increase of at least 20 mmHg with a standing systolic BP of at least 140 mmHg, accompanied by cardiac or neurological symptoms suggestive of acute end-organ stress. This study definition adapts the consensus criteria for orthostatic hypertension while adding the requirement for acute symptoms.

Data collection

Data was abstracted retrospectively from the electronic medical record. Variables included age, sex, body mass index, comorbidities, presenting symptoms, supine BP, standing BP, orthostatic systolic BP change, echocardiographic findings, cardiac catheterization results when available, inpatient management, outpatient recommendations, antihypertensive and diuretic medication exposure during hospitalization, documented medication timing when available, adherence to lifestyle and medical interventions, weight change, symptom status, recurrent hospitalization, and follow-up BP findings.

Outcomes

The primary descriptive outcome was the magnitude of orthostatic systolic BP increase at index presentation. Secondary outcomes included peak standing systolic BP, symptom patterns, HFpEF-related findings, inpatient management strategies, weight change, symptom improvement, resolution or persistence of orthostatic hypertension, recurrent hospitalization, and loss to follow-up.

Statistical analysis

Because of the small sample size and descriptive study design, formal hypothesis testing was not performed. Continuous variables were summarized using median values and ranges. Categorical variables were summarized as counts and percentages. Individual patient-level data were summarized in tabular form.

Ethical considerations

This retrospective review used existing clinical data and was conducted in accordance with institutional guidelines. The study was considered exempt from formal CAMC Institutional Review Board evaluation according to institutional policy.

## Results

Baseline patient characteristics and presenting symptoms

Seven hospitalized patients met the inclusion criteria. The median age was 56 years, with an age range of 47-60 years. Three patients were female. All patients had severe obesity, with body mass index ranging from 44.1 to 78.2 kg/m². Common comorbidities included hypertension, diabetes or prediabetes, obstructive sleep apnea, coronary artery disease, atrial fibrillation, chronic lung disease, prior cerebrovascular disease, and chronic HFpEF symptoms.

Presenting symptoms included dyspnea, chest pain, pulmonary edema, edema, syncope or near-syncope, confusion, altered mental status, dizziness, blurry vision, and stroke-like symptoms. Several patients had recurrent or longstanding cardiopulmonary symptoms before the recognition of orthostatic BP dysregulation. Baseline characteristics, index orthostatic BP values, and presenting symptoms are summarized in Table 1.

**Table 1:** Baseline characteristics and index orthostatic BP findings AF: atrial fibrillation; BMI: body mass index; BP: blood pressure; CABG: coronary artery bypass grafting; CAD: coronary artery disease; COPD: chronic obstructive pulmonary disease; CVA: cerebrovascular accident; DM: diabetes mellitus; DVT: deep vein thrombosis; HFpEF: heart failure with preserved ejection fraction; HLD: hyperlipidemia; HTN: hypertension; OSA: obstructive sleep apnea; PCI: percutaneous coronary intervention; SBP: systolic blood pressure; T2DM: type 2 diabetes mellitus

Case	Age/sex	BMI, kg/m²	Major comorbidities	Supine BP, mmHg	Standing BP, mmHg	ΔSBP, mmHg	Index symptoms
1	52/M	48.1	DM, HTN, atrial flutter, obesity, hypoxemic respiratory failure	156/77	189/86	+33	Dyspnea, edema, headache, dizziness, blurry vision, facial/arm numbness
2	47/F	47.0	HTN, depression, anxiety, smoking, prediabetes	154/84	194/80	+40	Confusion, chest pain, dyspnea
3	56/M	44.1	CAD, prior CABG/PCI, CVA, DM, HTN, HLD, HFpEF	139/64	160/115	+21	Chest heaviness, dyspnea, hypoxemia
4	58/M	59.3	OSA, COPD, chronic HFpEF, severe obesity	169/98	193/108	+24	Dyspnea, pulmonary edema
5	59/F	50.3	HTN, CAD, CVA, DVT, asthma, anxiety/depression, obesity	143/67	172/85	+29	Dyspnea, edema, oxygen desaturation
6	60/F	60.8	AF, T2DM, HTN, anxiety, osteoarthritis, obesity	131/77	171/112	+40	New AF, heart failure symptoms, dyspnea
7	52/M	78.2	OSA, HTN, DM, fatty liver, severe obesity	104/50	148/94	+44	Blackouts, tunnel vision, near-syncope

Orthostatic BP findings

**Figure 1:**
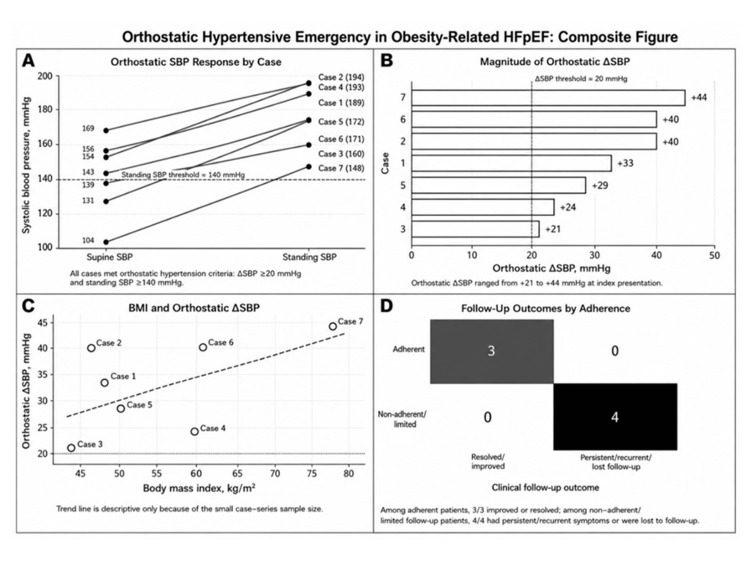
Orthostatic hypertensive emergency in obesity-related HFpEF Composite figure summarizing patient-level hemodynamic findings and follow-up outcomes among seven hospitalized patients with severe obesity, obesity-related HFpEF features, and symptomatic orthostatic hypertension. (A) Paired supine BP and standing SBP values by case, demonstrating a consistent standing-related rise in SBP. All patients met the study criteria for orthostatic hypertension, defined as an orthostatic SBP increase of at least 20 mmHg with a standing SBP of at least 140 mmHg. (B) Magnitude of orthostatic SBP increase by case, ranging from +21 to +44 mmHg at index presentation. The dashed vertical line indicates the ≥20 mmHg threshold for an exaggerated orthostatic pressor response. (C) Relationship between BMI and orthostatic SBP increase. The dashed trend line is descriptive only because of the small case series sample size. (D) Follow-up outcomes stratified by adherence pattern. Patients adherent to structured medical and cardiometabolic lifestyle interventions demonstrated improvement or resolution, whereas patients with limited adherence experienced persistent symptoms, recurrent hospitalization, or loss to follow-up. Data derived from the seven-patient retrospective observational cohort. This figure was created using Python (Python Software Foundation, Wilmington, Delaware, United States). BP: blood pressure; BMI: body mass index; HFpEF: heart failure with preserved ejection fraction; SBP: systolic blood pressure

At index presentation, supine systolic BP ranged from 104 to 169 mmHg, while standing systolic BP ranged from 148 to 194 mmHg. Initial orthostatic systolic BP increases ranged from 21 to 44 mmHg. The largest recurrent orthostatic systolic BP increase occurred in a non-adherent patient at follow-up, increasing from 109 mmHg supine to 188 mmHg standing. Patient-level supine and standing systolic BP values, orthostatic systolic BP changes, body mass index, and follow-up outcome patterns are shown in Figure 1.

Cardiac findings and management

All patients had preserved left ventricular ejection fraction. Several patients had evidence of diastolic dysfunction, elevated filling pressures, pulmonary congestion, post-capillary pulmonary hypertension, or elevated left ventricular end-diastolic pressure when invasive hemodynamic testing was available. These findings supported an obesity-related HFpEF phenotype.

All patients received individualized medical therapy, including antihypertensive therapy, diuretics for congestion, rate control or anticoagulation when indicated, and cardiometabolic lifestyle counseling. Patients were advised to monitor standing BP and pursue structured lifestyle changes, including dietary modification, weight loss, physical activity as tolerated, stress reduction, and medication adherence. Cardiac findings, inpatient management, and follow-up outcomes are summarized in Table 2.

**Table 2:** Cardiac findings, management, and follow-up outcomes ACE: angiotensin-converting enzyme; AF: atrial fibrillation; BP: blood pressure; ED: emergency department; EF: ejection fraction; HF: heart failure; HFpEF: heart failure with preserved ejection fraction; HTN: hypertension; IV: intravenous; LVEDP: left ventricular end-diastolic pressure

Case	Key cardiac findings	Inpatient/medical management	Lifestyle/weight outcome	Follow-up clinical outcome
1	EF 60-65%; obesity-related HFpEF phenotype; stroke workup performed	IV diuresis, aspirin, statin, antihypertensive therapy, oxygen	Persistent adherence challenges	Continued symptoms and difficulty maintaining lifestyle/medical adherence
2	EF 60%; recurrent hypertensive symptoms	Losartan, later metoprolol; BP monitoring	Approximately 10 kg weight loss	Orthostatic BP normalized; dizziness and chest pain resolved at follow-up
3	EF 65%; elevated cardiac output; LVEDP 17 mmHg; prior CABG/stent history	IV diuresis, losartan, statin, lifestyle intervention	Approximately 20 kg weight loss	Orthostatic BP changes resolved; cardiac symptoms improved
4	EF 60-65%; grade 2 diastolic dysfunction; pulmonary edema; chronic HFpEF	IV diuresis, metoprolol, lifestyle counseling	Semaglutide-associated weight loss but poor lifestyle adherence	Recurrent HF hospitalization; recurrent severe orthostatic hypertension
5	EF 55-60%; increased septal thickness; LVEDP 42 mmHg; post-capillary pulmonary HTN	IV diuresis, chlorthalidone, lisinopril, aspirin, magnesium	Persistent symptoms; adherence limited	Recurrent decompensation and ongoing HFpEF/obesity-related symptoms
6	EF 50-55%; persistent AF; non-obstructive coronary disease	Apixaban, aspirin, diltiazem, metoprolol, atorvastatin	Approximately 25 kg weight loss	Orthostatic BP changes resolved; cardiac symptoms significantly improved
7	No obstructive coronary lesions; LVEDP 33 mmHg	ACE inhibitor, beta-blocker, aspirin, standing BP monitoring	Non-adherent; lost to cardiology follow-up	Recurrent ED evaluation for vertigo/labile hypertension; lost to follow-up

Follow-up outcomes

Patients who adhered to lifestyle and medical recommendations demonstrated weight loss and improvement or resolution of orthostatic symptoms and BP abnormalities. In contrast, non-adherent patients experienced persistent symptoms, recurrent hospitalization, or loss to follow-up. Cohort-level demographic, hemodynamic, cardiac, and follow-up findings are summarized in Table 3.

**Table 3:** Summary of cohort-level findings BMI: body mass index; EF: ejection fraction; HF: heart failure; HFpEF: heart failure with preserved ejection fraction; SBP: systolic blood pressure

Variable	Finding
Number of patients	7
Median age, years	56
Age range, years	47-60
Female sex	3/7
BMI range, kg/m²	44.1-78.2
Median BMI, kg/m²	50.3
Supine SBP range at index presentation, mmHg	104-169
Standing SBP range at index presentation, mmHg	148-194
Initial orthostatic SBP increase range, mmHg	21-44
Recurrent maximum observed orthostatic SBP increase, mmHg	79
Preserved EF	7/7
HFpEF/obesity-related HF phenotype	7/7
Improvement/resolution among adherent patients	3/3
Persistent symptoms/recurrent hospitalization/lost follow-up among non-adherent patients	4/4

## Discussion

This retrospective observational study describes a symptomatic orthostatic hypertension phenotype among hospitalized patients with severe obesity and HFpEF features. All patients demonstrated an orthostatic systolic BP increase of at least 20 mmHg with a standing systolic BP of at least 140 mmHg, meeting the consensus criteria for orthostatic hypertension [1]. Unlike asymptomatic orthostatic hypertension, these patients presented with acute cardiac or neurological symptoms, including dyspnea, chest pain, pulmonary edema, confusion, stroke-like symptoms, and syncope or near-syncope. We therefore describe this phenotype as an orthostatic hypertensive emergency.

The findings are clinically relevant for several reasons. First, orthostatic hypertension is associated with adverse outcomes, including all-cause mortality, cardiovascular mortality, and cerebrovascular disease [2,3]. Second, obesity-related HFpEF is a growing clinical phenotype associated with hypertension, metabolic disease, inflammation, impaired vascular reserve, and exercise intolerance [4,5]. Third, orthostatic BP measurement is simple, low-cost, and readily available at the bedside. Failure to measure standing BP may miss clinically meaningful BP variability in patients with dyspnea, pulmonary edema, chest discomfort, dizziness, or neurological symptoms [6-9].

The mechanism of orthostatic hypertensive emergency remains uncertain and needs further study. We speculate potential contributors may be excessive sympathetic activation, impaired baroreflex buffering, vascular stiffness, endothelial dysfunction, volume redistribution, deconditioning, sleep apnea, pain, anxiety, and cardiometabolic inflammation. In obesity-related HFpEF, these mechanisms may be amplified by elevated filling pressures, impaired ventricular reserve, pulmonary hypertension, and reduced exercise tolerance [10-13]. Abrupt standing-related systolic BP elevation hypothetically could increase myocardial workload and contribute to symptoms even when supine BP appears only mildly elevated [14,15].

Clinical improvement among adherent patients suggests that management may require more than the escalation of antihypertensive therapy alone. Patients who achieved meaningful weight loss and adhered to cardiometabolic lifestyle intervention experienced improvement or resolution of symptoms and orthostatic BP abnormalities [16,17]. In contrast, patients with poor adherence had persistent symptoms, recurrent hospitalization, or loss to follow-up. These observations are hypothesis-generating and should not be interpreted causally, but they support the need for integrated HFpEF, obesity, hypertension, and lifestyle management.

There are currently no dedicated guidelines for orthostatic hypertensive emergency [18-20]. Standard hypertension and HFpEF management should be individualized, with careful attention to standing BP values, volume status, fall risk, symptoms, medication tolerability, and comorbid disease. Overly aggressive antihypertensive escalation based only on supine measurements may be problematic in patients with large postural BP variability. Conversely, failure to recognize severe standing hypertension may leave patients exposed to recurrent symptoms and potential end-organ stress.

This study has several important limitations. First, it was a retrospective, single-center observational study involving only seven patients, which limits generalizability, increases vulnerability to outlier effects, and prevents causal inference. Second, selection bias is likely because patients were identified only if they were hospitalized, evaluated by cardiology, and had orthostatic BP measurements documented during routine clinical care. Patients without measured or documented orthostatic BP values, patients unable to stand, and patients not referred for cardiology evaluation were not captured. Third, orthostatic BP assessment was not performed under a standardized prospective research protocol. Measurements were obtained using routine inpatient noninvasive automated oscillometric devices, but the device model, cuff size, exact duration of supine rest, repeat measurement frequency, time of day, dietary status, volume status, and timing relative to antihypertensive or diuretic administration were not uniformly documented. These factors may have influenced measured BP values and limit reproducibility. Fourth, follow-up was incomplete, and some patients were lost to cardiology follow-up, which limits the interpretation of symptom trajectories, recurrent events, and long-term BP outcomes. Fifth, apparent improvement among adherent patients should be interpreted as hypothesis-generating rather than causal because treatment adherence, weight loss, medication changes, comorbid disease control, and follow-up intensity were not randomized or standardized.

## Conclusions

Orthostatic hypertensive emergency may represent an under-recognized acute clinical phenotype requiring prospective validation in populations with severe obesity, cardiometabolic disease, and HFpEF. In this hypothesis-generating retrospective observational study, affected patients demonstrated marked standing-related systolic BP elevation accompanied by neurological or cardiac symptoms. Patients who adhered to structured medical and lifestyle interventions experienced improvement in symptoms and orthostatic BP patterns, whereas non-adherent patients had persistent symptoms, recurrent hospitalization, or loss to follow-up. Routine orthostatic BP assessment should be considered in hospitalized patients with obesity-related HFpEF symptoms, labile hypertension, unexplained dyspnea, syncope, chest discomfort, pulmonary congestion, or neurological symptoms. Prospective multicenter studies are needed to validate this phenotype and define optimal management.
